# Supplementary material for: Are carbon emissions trading and green financial instruments synergistic? -Comprehensive quantitative research based on content analysis
Source: PLoS One. 2024 Mar 7;19(3):e0298601. doi: 10.1371/journal.pone.0298601 (PMC10919601; doi:10.1371/journal.pone.0298601)
Supplement: S1 Data — (ZIP) [file pone.0298601.s001.zip › Data/地级市Z_F工作报告.zip!/║■─╧/╙└╓▌╩╨/└┤╫╘╣1⁄2╓┌║┼í╛┬φ┐╦╩2╛▌═°í┐_2012.docx]

各位代表：

现在，我代表市人民政府向大会作政府工作报告，请予审议，请各位市政协委员和列席人员提出意见。

一、2007年以来的工作回顾

肩负党和人民的重托，伴随时代前进的步伐，我们走过了极不平凡的五年。五年来，在市委的坚强领导下，全市上下坚持以科学发展观为指导，砥砺前行、开拓奋进，精诚团结、扎实苦干，胜利完成了市三届人大一次会议确定的目标任务。过去的五年，是永州历史上经济社会发展最快、城乡面貌变化最大、人民群众受益最多的时期之一。

科学发展开创新局面。经济增幅明显加快，2011年全市地方生产总值完成940亿元（预计数，下同）、比上年增长13％，总量是2006年的2．3倍；规模工业增加值完成260亿元、比上年增长22％，总量是2006年的4．2倍；社会消费品零售总额完成290亿元、比上年增长19％，总量是2006年的2．5倍；财政总收入完成70．5亿元、比上年增长31．7％，总量是2006年的3倍；金融机构存款余额851亿元、比上年增长18．3％，总量是2006年的2．28倍，贷款余额409亿元、比上年增长12．6％，总量是2006年的2．29倍。主要经济指标总量全部实现五年翻番。新型工业化强力推进，工业对经济贡献率达到43％，比2006年提高4．1个百分点，规模工业企业达到667家，其中产值过亿元企业150家，装备制造、农产品加工和矿产品加工三大百亿产业形成，电子信息、光伏、生物医药等新兴产业来势看好。农村经济持续较快发展，粮食生产连续五年丰收，现代烟草农业建设成效明显，农产品加工全省领先，国家和省级农产品加工龙头企业发展到31家，获得＂中国名牌产品＂2个、＂中国驰名商标＂4个、国家地理标志保护产品1个，市现代农业科技示范园晋升为国家农业科技园区和国家农业产业化示范基地。非公有制经济加速提质，发展活力进一步增强。城乡消费持续旺盛，文化旅游产业加速融合，商贸物流、新型消费业态、房地产等发展较快。节能减排扎实推进，关停和改造污染企业642家，建成污水处理厂11个、垃圾处理场9个，省定节能减排任务全面完成。＂绿色永州＂建设取得明显成效。

城乡建设展现新风貌。累计完成固定资产投资2636亿元，是前五年的4．47倍，实施重点项目2135个，其中投资过亿元项目130个，项目数量、投资总额、建设速度均创历史新高。立体交通网络加速形成。洛湛铁路建成通车，湘桂复线路基工程基本完工，铁路总里程达到461公里，新增244公里；邵永、道贺高速建成投运，高速公路已建在建488公里，二级公路新建在建796公里，全市公路密度是2006年的1．74倍；永州机场通航能力逐步提升。城镇带动功能显著增强。中心城区一体化步伐加快，永州大道建成通车，生态新城建设加速推进，中心城区建成区面积拓展到60．1平方公里、城区人口达到55万人，县城和中心镇建设迈出新步伐，全市城镇化率达到37．4％，比2006年提高7．2个百分点。2010年创建为省级卫生城市。农村基础条件大幅改善，改造新建农村公路1．58万公里，完成98座水库除险加固，新解决107万人口饮水安全问题，新开发整理耕地56万亩。涔天河水库扩建工程奠基，水库移民后扶工作取得实效。能源信息建设全面加强，新增水电装机76万千瓦，建成110千伏以上变电站24座、500千伏变电站2座，新增供电容量548．6万千伏安，行政村通电话率和互联网开通率分别达到90．6％和62．3％，广播电视综合覆盖率达到96．5％。

改革开放焕发新活力。承接、对接、合作力度空前，外资、外经、外贸成效显著。五年审批外资企业167家，实际利用外资16．2亿美元、内联引资636．3亿元、进出口总额7亿美元，分别为前五年的3．6倍、4．2倍和1．7倍，工业园区建成区面积150平方公里，已建在建标准厂房515万平方米，设置了海关、商检驻永州办事机构，招商引资工作五年都是全省先进，2008年被国家商务部确定为加工贸易梯度转移重点承接地，2011年被国家发改委确定为湘南承接产业转移示范区。对接东盟工作全省领先，对东盟出口额增长9．4倍，引进东盟企业4家，到东盟投资企业6家。与央企省企合作实现重大突破，长丰与广汽、南岭民爆与神斧集团成功重组，中国五矿、神华集团、海螺集团、北控集团、中交集团等与我市签订战略合作协议。金融创新迈出重大步伐，潇湘农村商业银行、江华农村商业银行、祁阳村镇银行挂牌营业，一批政府融资平台、中小企业信用担保公司、小额贷款公司成功组建，市城建投10亿元企业债顺利发行，跨境贸易人民币业务全面启动，顺利进入中国金融生态城市行列。省直管县财政体制改革全面实施，政府机构改革、集体林权制度改革、交通体制改革、医药卫生体制改革顺利推进。

社会事业取得新进步。大力实施教育强市、文化强市、科技兴市战略，社会事业发展的协调性持续增强。各类教育加快发展，建设合格学校280所，改造中小学危房132万平方米，新增城区学位7．1万个，永州四中顺利搬迁，湖南科技学院、潇湘技师学院建设加快，永州职院被评为国家级高职示范院校，永州工商职中和永州工贸中专被评为全国示范性职业中专。科技创新成果丰硕，组织实施省以上科技项目253项，申请专利3024件，新认定国家级高新技术企业24家。医疗卫生体系不断完善，基本药物制度全面推进，重大疫病防控扎实有效，市中心医院建成使用，8所县级医院标准化建设和189个乡镇卫生院改扩建完成。文化事业日益繁荣，文化惠民工程建设扎实推进，新建乡镇综合文化站177个、农家书屋3518家，新增国家文物保护单位26家、国家非遗项目5个。市体育中心被评为国家级全民健身活动中心。人口计生工作五年都是全省先进。国防后备力量建设得到加强，战备训练、现代营房建设进入全省先进行列。工青妇等群团组织作用充分发挥，＂五五＂普法全面完成，精神文明建设取得新成果。人事、统计、审计、质监、药监、物价、工商、民族宗教、外事侨务、贸促、对台、史志、档案、气象、水文、扶贫、人防、消防、残联、老龄、慈善、红十字会等工作都有新的进步。

民生保障达到新水平。加大民生投入，2011年全市民生支出占到财政总支出的70％，五年累计支出425亿元，是前五年的2．4倍。积极扩大就业，五年新增城镇就业26．7万人、农村劳动力转移就业46．9万人。完善社会保障体系，＂五项保险＂参保人数达495．2万人次，城镇居民养老、医疗保险实现全覆盖，新农保试点扩展到10个县区，新农合参合率超过98％，城乡低保实现应保尽保，社会救济救助水平逐步提高，住房公积金制度作用显现，新增廉租住房2．86万套，发放租赁补贴4．1万户，完成农村危房改造和倒房重建3．34万户。逐步提升城乡居民生活水平，2006年到2011年，城镇居民人均可支配收入由8725元提高到17132元，农民人均纯收入由2954元提高到5668元，城镇居民人均住房面积由31．4平方米提高到45平方米，每百户城镇家庭汽车拥有量由0．8辆提高到5．7辆，每百户农民家庭摩托车拥有量由8．1台提高到20．5台。安全生产四年获全省先进。依法信访得到加强，信访积案化解成效明显，维稳保安能力得到提升，命案侦破连续四年、命案积案侦破连续五年排名全省第一，追逃＂清网行动＂、社会管理创新和公众满意度测评位居全省前列，社会大局保持和谐稳定。

依法行政迈出新步伐。各级政府认真执行人大及其常委会的决议、决定，自觉接受政协的民主监督，五年共办理市人大代表议案和建议、批评、意见977件，市政协委员提案991件，办复率均为100％。市政府重大决策及时向市委报告，提请市人大审议，主动与市政协协商，市政府常务会议邀请市委、市人大、市政协、军分区领导参加，并请群众代表列席。制定出台《重大行政决策程序规则》，建立健全行政决策听证、行政裁量权基准、政务信息公开和规范性文件管理等制度。深化行政审批制度改革，加强政务服务中心建设和管理，推行并联审批、全程代理、打捆收费、限时办结和园区封闭管理，优化经济环境和机关效能建设、市政府行政程序建设、政务公开工作连续四年全省先进。强化政府绩效考核、行政首长问责和重点项目目标管理，进一步增强了政府工作的推动力和执行力。

不平凡的五年，成就了不平凡的业绩，永州发展已经站在新的历史起点上。过去五年，许多经验弥足珍贵。

－－过去五年，我们立足赶超，抢抓机遇，赢得了发展先机。坚持未雨绸缪、超前谋划，在解放思想中捕捉机遇，在形势变化中用好政策，紧紧抓住中央扩内需、保增长的机遇，中央和省里促进转型发展、建设＂两型＂社会的机遇，省委、省政府提出敞开南大门、对接粤港澳、加快湘南开放开发，把永州建成全省产业承接示范区、对接东盟桥头堡的机遇，大干快上，只争朝夕，建成了一批事关永州全局和长远发展的大项目好项目，办成了一批多年想办而没有办成的大事好事。

－－过去五年，我们攻坚克难，创新求实，破解了发展难题。面对国际金融危机带来的巨大冲击，化危为机，变压力为动力，紧紧围绕保增长、保民生目标，制定实施了抓项目、增投入、促消费等政策措施，全市经济逆势上扬，实现弯道超车，2008年至2010年，年度主要经济指标增幅位居全省前列；面对百年不遇的冰冻灾害和严重旱涝灾害带来的不利影响，全市上下万众一心，全力以赴救灾安民，把灾害损失降到了最低程度；面对宏观经济环境复杂变化带来的诸多困难，坚持用改革的办法、市场的手段化解矛盾问题，用创新的思路、求实的精神推动加快发展，始终保持了经济发展的良好势头。

－－过去五年，我们主攻重点，狠抓关键，谋求了发展优势。按照市委的总体部署，突出以项目建设为统揽，通过实施一批重大项目，扩大资金投入，优化产业结构，提升发展水平；突出以交通建设为重点，加快推进基础设施建设，为经济社会发展提供强力支撑；突出以工业发展为主导，大力实施＂五百工程＂、＂四大战略＂，以新型工业化带动现代农业、提升第三产业；突出以生态新城建设为龙头，强力推进中心城区一体化，积极发展县城和中心镇，促进城乡统筹发展；突出以承接对接为抓手，全面扩大开放引进，大力发展开放型经济。全市优势条件逐步发挥，发展特色日益显现。

－－过去五年，我们以人为本，民生优先，践行了发展宗旨。始终坚持发展为了人民，发展成果全民共享。加大民生投入，在财政比较紧张的情况下，多方筹措资金，确保民生之需；加快项目推进，一批事关民生的重大项目相继建成，人民群众就医难、就学难等问题逐步得到解决；坚持走绿色发展之路，加强生态建设，推进节能减排，全市生态环境质量保持全省前列。

各位代表，过去的五年，历程极不平凡，成绩来之不易，我们深切感到，永州的发展变化，靠的是市委的坚强领导，靠的是历届班子打下的坚实基础，靠的是全市人民的不懈努力，在此，我代表市人民政府，向所有关心、支持、参与永州建设发展的同志们、朋友们，表示衷心的感谢！

在看到成绩的同时，我们也清醒地认识到，经济社会发展过程中仍存在不少困难和问题，主要是，经济总量不够大、结构不够优、质量不够高，转变发展方式任重道远；一些群众生活还比较困难，保障和改善民生任务艰巨；社会转型过程中还存在不少矛盾，社会管理水平有待提高。对此，我们必须高度重视，认真加以解决。

二、今后五年的主要目标和任务

未来五年，是我市全面完成＂十二五＂规划、加快建设全面小康的关键时期，是转变发展方式、推动赶超崛起的攻坚时期，也是充满发展机遇、可以大有作为的黄金时期。纵观国际宏观环境，既要看到国际金融危机以后，世界经济形势总体上仍将严峻复杂，世界经济复苏的不稳定、不确定性上升，又要看到当今世界正处在大变革、大调整之中，科技创新方兴未艾，经济结构深度调整，产业转移步伐加快，为我们扩大开放合作、建设＂两型＂社会提供了难得机遇。分析国内经济走势，既要看到国家继续加强宏观调控，经济发展面临不少新情况新问题，又要看到我国经济发展长期向好的趋势没有改变，重大政策、重大项目投资主要转向扩大消费、改善民生、完善基础设施、扶持中西部地区发展，有利于我们争取更多的政策支持。立足现实基础条件，既要看到我市经济欠发达的市情没有根本改变，全省及周边城市迅猛发展，给我们加速提质发展带来竞争压力，又要看到我市经济发展前景很好，内生动力显著增强，湘南承接产业转移示范区建设上升到国家层面，省委省政府＂四化两型＂、＂两个加快＂战略深入实施，为我市发展提供了强大引擎，增添了强劲动力。我们一定要准确把握形势，坚定发展信心，始终保持昂扬向上、奋发有为的精神状态，始终保持脚踏实地、真抓实干的工作作风，在新一轮发展中抢占制高点，赢得主动权，争当排头兵。

全市经济社会发展总体要求是：高举中国特色社会主义伟大旗帜，以邓小平理论和＂三个代表＂重要思想为指导，深入贯彻落实科学发展观，按照省委省政府＂四化两型＂、＂两个加快＂的战略部署，坚持科学赶超、开放崛起、富民强市，以建设承接产业转移示范区为总抓手，着力强基础、兴产业、建新城、重环保、惠民生，努力打造现代工业城市、湘粤桂省际区域中心城市、生态宜居城市、知名旅游目的地城市，为全市人民的幸福生活而努力奋斗。

全市经济社会发展主要预期目标是：地方生产总值年均增长13％以上，规模工业增加值年均增长20％以上，固定资产投资年均增长25％以上，社会消费品零售总额年均增长19％以上，财政总收入年均增长15％以上，城乡居民收入年均增长13％左右。节能减排完成省定目标任务。人口自然增长率控制在7．2‰以内。城镇化率达到46％以上。

全市经济社会发展的战略举措是：

（一）发展大工业，加快产业转型。实现富民强市，核心在工业，希望在工业，必须坚持工业主导战略不动摇，以工业的持续快速发展，推动产业转型升级，提升整体经济实力。

壮大规模做强工业。加快工业发展，必须坚持加速提质并举，壮大促优并重。狠抓大项目，积极向上争取，扩大招商引资，强化资源整合，开发新上一批大项目好项目，力争五年内实施投资1亿元以上工业项目200个以上。发展大企业，壮大企业主体，延伸产业链条，推动联合兼并，着力培育一批大企业大集团，力争五年内建成年产值过亿元企业300家以上，全力将长丰汽车、零陵烟厂、永州火电厂、达福鑫电子、湘龙铜业打造成永州工业的＂航空母舰＂。培育大产业，着力改造提升传统产业，培育壮大新兴产业，做大做强优势产业，力争五年内建成江华稀土、零陵锰业等11个年产值过百亿的支柱产业，其中先进装备制造、电子信息和光伏、矿产品加工、农产品加工、外向型加工贸易等五大产业年产值突破300亿元。争创大品牌，力争五年打造15个以上省级名牌。构建大格局，打破行政壁垒，优化区域布局，在全市构建四大经济板块，即湘江千亿＂两型＂产业带、环阳明山绿色经济圈、蓝宁道新加工贸易走廊、湘粤桂边界民族经济区，形成特色鲜明、优势突出的发展格局。

培育特色做优农业。坚持以农民增收为根本目的，以现代农业为发展方向，放大优势，做靓特色，创优品牌，推动我市由农业大市向农业强市跨越。大力推进规模农业基地建设，全面实施粮食千亿产能工程和粮油百亿产业工程，着力建好450万亩优质稻、80万担优质烤烟、180万亩无公害蔬菜、320万亩油茶、150万亩水果、100万亩工业原料林、1000万头生猪、50万头肉牛、富硒香型农业等规模农业基地，打破地域界限，发挥优势条件，建设一批特色农产品产业带。大力发展农产品加工龙头企业，围绕粮油、果蔬、药材、畜禽开展精深加工，创建一批农业标志性企业和主导品牌，力争把永州建设成为省内领先、全国知名的农产品加工基地。大力实施现代农业示范园区创建工程，加大资源整合和资金投入，着力将市现代农业科技示范园建成全国一流的农业科技园区、农业产业化示范基地和新农村建设样板区，创造条件，建设一批县区现代农业示范基地和示范园区。大力加强农业综合服务体系建设，积极探索农村土地流转、水利建设管理，以及科技、金融、信息服务新机制，不断完善农村市场体系，培育壮大农民经纪人队伍和农民专业合作组织。

提升水平做大第三产业。加快实施文化强市战略，推进文化旅游融合发展，做大做强文化旅游产业。全面完成旅游＂1010＂工程，着力建好一批重点旅游景区和高星级旅游饭店，打造几条精品旅游线路。加大宣传推介力度，着力抓好祭舜大典、江永女书节、阳明山＂和＂文化旅游节暨杜鹃花会、瑶族盘王节、周敦颐国际理学文化节、柳文化节、孝文化节、东安武术节等大型文化旅游活动。大力发展新闻出版、文化创意、广播影视、文娱演艺等文化产业，加快建设将帅影视城、市文化艺术中心等一批标志性文化工程。加快发展大商贸大物流＂1020＂工程，突出抓好国际航空物流园、蓝宁道新保税物流中心、永州商务会展中心等一批重大项目，推动各种新型服务业态发展壮大。

（二）推动大开放，构筑承接高地。全力以赴抓好国家级承接产业转移示范区建设，突出＂产业承接、绿色引领、文化创新＂三大特色，以科学承接、互利承接、绿色承接为导向，以体制机制创新为动力，拓展开放领域，提升开放水平，努力把永州建成产业承接新平台、区域合作引领区、加工贸易集聚区、转型发展试验区，争当全省对外开放的排头兵。

突出承接重点。在空间布局上，着力构建＂一区一廊五带＂，即凤凰园经济开发区，蓝宁道新加工贸易走廊，湘江千亿＂两型＂产业带、二广高速、泉南高速、厦蓉高速和道贺高速沿线发展带。在产业建设上，着力引进发展汽车及零部件产业、电子信息和光伏产业、矿产品和农产品精深加工业、生物医药产业、外向型加工贸易产业和文化旅游产业。在发展路径上，着力实现六个转变，即从承接低端产品生产企业为主向承接高端产品生产企业为主转变，从承接配件生产企业为主向承接总装生产企业为主转变，从承接单个企业向承接产业集群转变，从承接劳动密集型企业为主向承接劳动、资金、技术密集型企业为主转变，从以蓝宁道新加工贸易走廊承接为主向全市范围全面承接转变，从以承接沿海产业转移为主向承接全国、东盟乃至国际产业转移转变，加快构建全方位、宽领域、多层次的产业承接格局。

夯实承接平台。加快交通平台建设，着眼对外大通畅、对内大循环，开展交通建设三年大会战，全力打通南下北上、东进西拓大通道，尽快形成高速公路、铁路、航空、水运四位一体的现代立体交通网络。加快园区平台建设，力争五年内凤凰园经济开发区建成国家级经济开发区、蓝宁道新加工贸易走廊建成国家级出口加工区，技工贸收入双双突破千亿元，市现代农业科技示范园、基础较好的县区工业园建成产值过百亿园区。五年全市新增标准厂房800万平方米。采取委托管理、投资合作等形式，与沿海城市、企业、东盟国家合作共建产业园区，推动优势互补、互利共赢；引导各县区合作共建产业园区，整合资源、联动发展。加快通关平台建设，设立永州海关和检验检疫机构，建成永州铁海联运口岸，开通永州至广州、深圳、湛江等地的＂五定＂班列，建设面向东盟的国际航空物流港，争取在广西钦州设立永州对接东盟的出海港口基地。

创优承接环境。着力推进观念创新。敢于打破常规，勇于开拓创新，以更加开放的思维、更加广阔的胸襟、更加高远的眼光，确定示范区发展目标，编制示范区建设规划。着力推进政策创新。用好用活先行先试政策，紧密结合永州实际，制定出台推进示范区建设的优惠政策，构建强有力的政策支撑体系。着力推进体制机制创新。深化行政管理体制、市场体系、金融服务、土地管理、户籍制度、人才引进等方面的改革，加强法制、诚信和人文环境建设，为示范区建设提供体制机制保障。

（三）培育大城市，打造宜居之都。紧紧抓住国家促进中部崛起规划把永州作为100－200万人口的大城市进行培育的历史性机遇，突出规划引领，壮大城市规模，提升发展水平，努力把永州建设成为国家历史文化名城、全国知名旅游目的地城市、国家园林城市、国家森林城市、国家卫生城市。

优化空间发展格局。中心城区要以零冷两区一体化为核心，以生态新城建设为重点，尽快拉开＂一圈两轴两带三组团＂的基本框架，到2016年，建成区面积拓展到100平方公里、人口突破80万。按照＂生态理念建城、基础设施连城、高端产业融城、古韵新貌靓城＂的思路，加快生态新城建设步伐，力争三年出骨架、五年成规模，构建零陵古城区、冷水滩现代城区和生态新城交相辉映的中心城区新格局。依托中心城区，以祁阳－冷水滩－东安为横轴，以冷水滩－零陵－双牌为纵轴，以两区和三个县城为节点，构建＂T＂字型中心城市群，南部逐步发展以六个县城为节点的副中心城市群。

提升城市建设品位。切实增强集聚辐射带动功能，加快推进中心城市高速外环线、城际快速通道和阳明大道、潇湘大道等城区主干道建设，规划建设一批城市综合体。注重城市功能的配套性、整体性和服务性，重点推进一批城市公交、市政设施、住房保障、教育卫生、商贸服务和休闲娱乐设施建设，基本完成城市棚户区、城中村、背街小巷和农贸市场改造。提升城市管理水平，加快构建网络化、数字化、精细化的长效管理机制，让城市更加净化、美化、亮化。

彰显生态人文特色。围绕打造生态宜居城市，突出＂两型＂引领，坚持环保优先，严格建设项目环评审批，严格控制＂两高一资＂项目，加快实施湘江潇水流域生态环境综合治理工程、垃圾污水处理工程、森林城市建设工程，着力抓好湘江两岸风光带建设、＂三边＂植树造林、20万棵大苗进城和部门联村建绿，新建改造一批城市公园和公共绿地，建好一批生态旅游乡镇、新农村建设示范片，加强山水景观建设和自然生态保护，充分展现山水洲城形象，努力建设绿色永州。围绕创建国家历史文化名城，加快构建历史文化名城保护体系，加强零陵古城和历史文化街区、文物古迹、古镇、古村落、古民居的保护开发，新建一批具有历史底蕴、文化内涵和地域特色的标志性建筑，推动人文资源与文化旅游、城市建设有机融合，全面提升城市文化价值和文化品位。

（四）主攻大交通，强化基础支撑。基础设施建设事关长远、影响全局，要始终坚持打基础、增后劲不动摇，以交通建设为重点，不断完善基础设施体系，全面提升支撑保障能力。

加快推进交通建设。围绕打造湘粤桂省际交通枢纽，着力构建以＂两纵两横＂高速公路网、＂井＂字型铁路网、国际航空港、湘江航运为主的立体交通网络。全面建成湘桂铁路复线和永蓝、厦蓉两条高速公路，打通省际断头路，争取洛湛铁路扩能项目、永郴铁路、桂永郴赣铁路和永郴、祁道两条高速公路开工建设。加强永州机场改造维修，启动永州机场搬迁前期工作，开辟10条以上空中航线。实施湘江航道改扩建工程，在永州境内形成千吨级航运能力。积极开展湘桂运河建设前期工作。全力构筑以干线公路、城际快线、农村公路为主的市域循环网络，打造北五县区半小时经济圈、中心城区至南部六县两小时经济圈，以及南六县区域间一小时经济圈。

加快推进能源建设。全力支持神华集团永州火电项目早开工、早建设、早出成效，着力抓好涔天河、湘祁、白滩河等骨干水电站建设，大力发展风能、太阳能、生物质能和沼气等可再生能源，切实加强电网建设和改造，扎实推进管道天然气工程和能源储备库建设，构建安全可靠的能源供应和保障体系。

加快推进农田水利建设。加大工作力度，按时优质完成涔天河水库扩建工程。规划建设何仙观、晨光、毛俊、金钩挂、芦江水库等一批重点水利工程。推进病险水库除险加固、山洪地质灾害防治、城市防洪工程建设，加强中低产田改造、土地综合整治、中小河流治理、大中型灌区及配套建设，提高综合防灾减灾能力。加快推进水资源管理体制、水利投融资体制、水利工程建设和管理、基层水利服务体系和水价等五项改革，切实增强水利在经济社会发展中的支撑保障作用。

加快推进数字永州建设。积极推动＂三网融合＂，切实加强电子商务、电子政务、物联网、地理数据、公共应急指挥中心、网上供销社等公共信息服务平台建设。大力发展电子信息产业，扶持发展新型信息服务业。坚持以集约共享为方向，促进信息技术在经济社会各领域的广泛应用，推动政务、商务、生产、生活、社会管理的信息化。

（五）促进大和谐，建设幸福永州。顺应全市人民过上美好生活新期待，加强社会建设，创新社会管理，让全市人民学有优教，劳有多得，病有良医，老有善养，住有宜居，共建共享幸福永州。

大力实施城乡居民收入倍增计划。推动全民创业，大力扶持中小企业发展，突出抓好高校毕业生、农村转移劳动力、城镇零就业家庭等群体的就业创业，逐步提高最低工资和劳动报酬。深化农村产权制度改革，赋予农民更加充分的财产权利，扩大农民财产性收入。加大民族政策落实力度，抓好扶贫开发和水库移民后扶工作，增强少数民族地区、贫困地区、山区、库区群众增收致富能力。

加快构建惠及全民的社会保障体系。完善城镇职工基本养老保险制度，推动城乡居民养老保险全覆盖，实现＂人人享有基本养老保障＂的目标。全面推进城镇居民医保、新型农村合作医疗、城乡医疗救助制度建设，实现＂人人享有基本医疗保障＂的目标。进一步完善城乡低保制度，不断提高救济救助水平，实现＂城乡困难群众人人享有基本生活保障＂的目标。加快构建住房保障体系，大力推进廉租房、公租房建设和棚户区改造，继续实施农村危房改造和安居工程，扩大住房公积金覆盖面，实现＂居者有其屋＂的目标。

全面发展各项社会事业。加快建设教育强市。大力发展学前教育，加强公办幼儿园建设，支持民办幼儿园提供普惠性服务。巩固提升义务教育，切实抓好农村合格学校和城区学位建设。加快普及高中教育，把永州一中、永州四中打造成省内名校。大力发展高等教育和职业教育，启动建设潇湘大学，支持永州职业技术学院、潇湘技师学院做大做强，推进县区职教中心建设。支持鼓励社会力量办学，促进民办教育健康发展。加快建设创新永州。完善以政府为主导、企业为主体、市场为导向、产学研相结合的技术创新体系，促进科技成果加快转化为现实生产力。大力培育创业创新文化，保护知识产权，鼓励发明创造，提高全民科学素养。切实做好人才的培养、引进和使用工作，五年引进2000名以上优秀人才。推动文化大发展大繁荣。以社会主义核心价值体系为引领，推进思想道德建设，健全社会诚信体系，培育积极向上、健康文明的社会风尚。加快文化体制改革，推进文化惠民工程建设，构建布局合理、功能完善、覆盖城乡的公共文化服务体系。繁荣文艺创作，推出文化精品，培育一批龙头文化企业。深入推进医药卫生体制改革，健全完善公共卫生和医疗服务体系、医疗保障体系、药品供应保障体系，提高应急处置突发公共卫生事件的能力和水平。加强人口和计划生育工作，稳定低生育水平，提高人口素质。深入开展全民健身运动，提高竞技体育水平。高度重视妇女、儿童事业，切实做好老龄工作，不断完善残疾人服务和保障体系。促进民族团结进步、繁荣发展。切实加强＂双拥＂工作，大力支持国防和驻永部队建设，完成县区人武部营房建设任务，提高国防后备力量建设质量和水平，抓好人民防空工作。加快推进法治永州建设，全面完成＂六五＂普法任务。

加强和创新社会管理。创新社会管理体系，建立党委领导、政府负责、社会协同、公众参与的社会管理格局。健全社会管理机制，探索建立科学有效的利益协调、诉求表达、矛盾调处、权益保障、治安防控和民主自治机制，加强源头防范，化解社会矛盾。推进社会管理重心下移，建立健全以社区自治组织为主体、社区居民广泛参与的新型社区管理和服务体系。加强和完善信息网络管理，提高管理虚拟社会的能力和水平。坚持以群众工作总揽信访工作，健全群众权益维护机制，切实解决群众合理诉求。加强社会治安综合治理，严厉打击各种违法犯罪。高度重视安全生产，坚决遏制重特大安全生产事故发生。加强应急管理和防灾减灾体系建设，提高应急处置、事故救援和抗灾救灾能力。

三、2012年的工作重点

今年是新一届政府履职的第一年，开好局、起好步至关重要。全年经济社会发展主要预期目标是：地方生产总值增长12．5％，规模工业增加值增长23％，固定资产投资增长25％，社会消费品零售总额增长19％，进出口总额增长25％，财政总收入增长15％，城乡居民收入增长12％，综合经济实力实现＂三个一千＂，即地方生产总值、规模工业总产值、金融机构存款余额均突破1000亿元。节能减排完成省定目标。

（一）着力抓好工业发展。坚持项目兴工，通过实施一批大项目好项目，带动和促进工业发展，力争全年开工新建投资500万元以上工业项目300个，竣工投产100个。积极协调化解各类矛盾，力促去年竣工的一批工业项目尽快投产达效。狠抓企业挖潜增效，确保产品有市场、带动能力强的骨干企业满负荷生产，重点抓好零烟、湘纸结构调整，力促长丰CP2轿车三季度建成投产，稳步扩大湘龙铜业、奔腾彩印等企业生产规模。按照＂一企一策＂办法，扶持困难企业恢复发展生产。做好规模企业入统工作，确保全年新增规模工业企业70家以上。全面开展＂项目建设年＂和＂企业服务年＂活动，市里确定一批重大项目和重点骨干企业，由市级领导和相关部门实行定点联系、挂牌服务。

（二）着力抓好农业农村经济。落实国家强农惠农富农政策，加快农业科技进步，促进农业增产、农民增收、农村发展。扎实推进规模农业基地建设，耕地总量稳定在491．7万亩，基本农田总量稳定在435万亩，粮食播种面积稳定在824万亩，烤烟种植28万亩、收购75万担，水果低改扩园30万亩，新增造林面积48万亩。新建100个标准化规模养殖场，新建农村沼气池6000口、大中型沼气工程203处，推广先进适用农机具5万台（套）。提质发展农产品加工业，年内新增销售收入过亿元企业4家，新培育省级以上名牌产品3个。大力发展农民专业合作组织，新增专业合作社100家，创建11个省级示范社。抓好涔天河水库移民搬迁安置工作，启动200个新农村建设示范村和11个示范片建设。加快推进农村集体土地确权登记发证。实施乡村清洁工程，鼓励引导农村相对集中建房。实施高标准农田示范工程建设，推进涔天河耕地后备资源开发。落实国家新十年扶贫开发纲要，全面启动新一轮扶贫开发，全年减少贫困人口3万人。市现代农业科技示范园要建设一批观光农业、高效农业基地和乡村旅游景点，新引进一批农产品加工龙头企业。

（三）着力抓好城镇建设和管理。精心制定中心城区战略发展规划，全面完成控制性详规和专项规划编制，加快生态新城建设步伐，抓紧实施永州大道景观带、湘江两岸风光带、玫瑰湾国际生态旅游度假区、卷烟物流配送中心、国际航空物流园、影视文化中心等项目。加强市政基础设施配套，加快九嶷大道、梧桐东路、零陵区沿江大道以及零陵工业园、火车站配套路网建设，打通舜皇路、银象路、潇湘大道等断头路，改造珍珠路、梅湾路，开工建设湘江东路、湘江西路、城南大道和城南大桥，加快城中村、河西旧城区、湘江西路棚户区以及背街小巷改造，全面提升建成区配套功能和水平，严厉打击违法建设行为。大力实施县城建设＂八个一＂工程，突出抓好路网管网配套、污水和生活垃圾处理设施、城区公园和绿地建设，全面开展卫生县城、文明县城创建活动。切实抓好乡镇所在地的绿化美化和环境卫生，着力打造一批标准化示范乡镇，以此带动新农村建设，促进城乡统筹发展。加强和创新城市管理，推动＂五城同创＂工作向纵深开展。

（四）着力抓好现代服务业发展。加快发展生产性服务业，突出抓好蓝宁道新保税物流园、潇湘汽贸城、粮油综合批发大市场、永州新商城、矿产品交易中心等重点项目建设，大力发展连锁经营、物流配送、仓储超市、电子商务、网上购物等新型业态，年内要在冷水滩区河东、河西各开工建设一个城市综合体。全面提升商贸流通、邮政快递、体育健身、休闲娱乐、家政养老、社区服务等生活性服务业，大力发展数字媒体、数字出版、文化创意等新兴文化服务业。发展壮大旅游产业，加强旅游市场开发和旅游配套设施建设，推进九嶷山舜帝陵创5A和柳子景区、阳明山国家森林公园创4A工作，完成李达故居、周敦颐故里、新田谈文溪村创3A旅游区工作，年内冷水滩、零陵、道县、宁远、祁阳力争各建成一家五星级宾馆，其它县力争各建成一家四星级宾馆。

（五）着力抓好基础设施建设。抓紧交通项目实施，年内永蓝高速、厦蓉高速、二广高速双牌连接线、东安至广西全州、省道323新田至宁远、省道324蓝山段改造工程等建成通车，加快湘桂铁路复线、省道325道县祥林铺至江永龙虎关、国道207东安段和江华段、国道322零陵改线工程、国道322祁阳绕城公路、省道323宁远绕城公路、祁阳至冷水滩一级公路、江华白芒营至石碧塘等项目施工进度，新开工建设厦蓉高速宁远互通至九嶷山、祁阳木梓圩至金洞、新田邬下至金鸡岭、国道207零陵至双牌一级公路、东安芦洪市至小江口、省道326江华白芒营至码市分水岭等项目，抓紧做好永郴铁路、桂永郴赣铁路、祁道高速公路、永郴高速公路、冷东快速通道和萍岛至归阳航道改造前期工作。启动永州机场搬迁前期工作。加强水利设施建设，全力抓好涔天河水库扩建工程，加快推进52座小一型和116座小二型水库除险加固，全面完成25万人农村饮水安全工程，抓好11个中小河流治理、6个城市防洪工程和6个小农水重点县建设，加强清淤防渗和渠系配套。加强耕地保护和矿产资源开发秩序整治，坚决制止耕地抛荒，完成河道采砂整治任务。抓好一批电源电网建设，争取永州火电项目一期工程今年四季度开工建设，加快湘祁和五里牌电站、江华风电等项目建设进度，开工建设双牌、江永、蓝山风电项目，扩建1座220千伏变电站，新建和续建3座110千伏变电站，抓好14座35千伏变电站建设，加大城乡电网改造力度，推进新一轮农网升级改造工程。实施石油储备库和城市管道天然气工程。加强各类信息传输网络建设，推进电信网、广播电视网和互联网＂三网融合＂。

（六）着力抓好承接产业转移示范区建设。坚持高起点规划，大力度推进，上半年完成示范区区域建设规划、产业布局规划的编制工作，制定出台相关扶持政策。加大招商引资力度，全年引进各项资金增幅不低于全市固定资产投资增幅，实际利用外资5亿美元以上，内联引资达到250亿元。突出抓好引资重点，紧紧围绕做大做强先进装备制造业、电子信息和光伏产业、矿产品和农产品加工业、加工贸易型产业、文化旅游产业和清洁能源产业，承接沿海产业转移，加强与央企省企和东盟合作，促进产业集群集聚发展。提升发展工业园区，全年新增园区面积20平方公里以上，新建标准厂房150万平方米以上，新引进落户规模工业企业100家以上，今年对工业园区建设要制定严格的目标管理制度和考核奖惩办法。加强通关平台建设，争取省里支持我市在北部湾设立湖南对接东盟出海港口基地，建成蓝山公路口岸。完成海关大楼主体工程。降低门槛，拓宽领域，加快发展非公有制经济。

（七）着力抓好财税金融工作。加快财源建设，整合财政资金，全力支持承接产业转移示范区建设，重点扶持财税贡献大的企业加快发展；强化财税征管，进一步理顺市区税收征管体制，完善收入组织调度、非税收入征缴和目标考核机制，严格规范执收执罚和减免缓征行为；牢固树立过紧日子的思想，调整和优化支出结构，切实保障重点支出；加强财政监管，防范和化解债务风险。加大信贷投入，全年新增贷款80亿元，力争金融机构存贷比达到50％；完善金融体系，年内新引进股份制商业银行1家，新组建村镇银行1至2家，新增小额贷款公司和融资性担保公司2至3家，新组建双牌、东安、祁阳、蓝山等4家农村商业银行，以潇湘农村商业银行为基础，着力打造一家资产过百亿元的区域性银行，全面消除金融服务空白乡镇，做大做强城建投、交建投等融资平台；鼓励和推动上市融资，力争1至2家企业上报国家证监会发审委审批；深入开展金融创安活动，加强社会信用体系建设，严厉打击非法集资活动，切实优化金融生态环境，力争创建1至2个省级金融安全区。

（八）着力抓好民生保障。重点实施十大惠民工程。一是就业和社会保障惠民工程。加快省级创业型城市建设，全年开展职业技能培训14万人次，新增城镇就业5万人，稳定农村劳动力转移就业130万人以上。五项社会保险参保人数达到500万人次，城镇居民养老保险参保人数达到15万人、城镇医疗保险参保人数达到168万人，新农合参合率稳定在95％以上。二是科技惠民工程。实施省级以上科技项目40项，申请专利800件，授权专利300件，启动实施科技特派员农村科技创业行动。三是教育惠民工程。认真落实学前教育三年行动计划，年内建好20所公办幼儿园，扶持40所普惠性民办幼儿园。加快6所特殊学校建设，完成65所合格学校建设任务，继续加强城区学位建设，开展农村义务教育营养改善计划试点。加快永州职业技术学院、潇湘技师学院建设，创建2个省级以上职业教育改革发展示范学校、2个省示范性县区职教中心。加快推进以校车为重点的学生安全工程建设。四是文化惠民工程。加快市文化艺术中心、中国将帅影视基地、凤凰影视城、萍洲文化广场等文化项目建设，承办好全省公祭舜帝大典活动，建好1684家农家书屋，全面推进＂三馆一站＂等公共文化阵地免费开放工作，加强网吧整治和管理。五是卫生惠民工程。加快市中心医院全科医生培训基地、芝山医院、市妇幼保健院等项目建设，完成3所县区医院、11所县区卫生监督所、5所县区急救中心、4所乡镇卫生院项目建设任务，实现全市村卫生室和乡村医生全覆盖，积极探索公立医院改革试点。六是全民健身惠民工程。规划建设一批城市社区体育设施，完成第二批新农村建设农民体育健身工程项目。七是安居惠民工程。完成保障性安居工程建设和农村危旧房改造任务，建设廉租住房7100套、公共租赁住房5100套，城市棚户区改造2600户，林区棚户区改造2626户，垦区危旧房改造2349户，农村危旧房改造8000户。八是计生惠民工程。符合政策生育率达到81．5％以上，人口自然增长率控制在7．5‰以内，出生性别比有所下降。九是生态惠民工程。大力开展绿色永州五年行动、污染治理专项行动和节能减排全民行动。十是维稳保安惠民工程。强化信访工作责任，严格落实接访、处访、下访制度，维护正常信访秩序。加强特殊群体的管控，高度重视禁毒工作。深入开展＂＂打黑除恶＂、打＂两抢一盗＂、＂打四黑除四害＂、＂命案必破＂和＂计量六进＂活动，确保人民群众生命、财产和食品、药品安全；加强基层安全生产，建成25个省、市级安全生产示范乡镇（社区）；完善社会治安防控体系，加强城市技术视频防控网、街面巡逻防控网、城乡社区防控网、单位和行业场所防控网、区域警务协作网、虚拟社会防控网建设，推进基层平安创建活动；组织开展＂三强行为＂专项整治行动，营造良好的建设施工环境。

各位代表，承载光荣使命，肩负历史重任，对政府工作提出了新的更高要求。各级政府及其工作人员要倍加珍惜来之不易的发展局面，全力凝聚科学赶超的智慧力量，朝夕勤勉，永不懈怠，努力建设人民满意的法治政府、责任政府、服务政府、廉洁政府。坚持依法行政。切实增强依法行政意识，认真执行人大及其常委会的决议、决定，主动报告工作，依法接受监督，全力支持政协履行政治协商、民主监督和参政议政职能，广泛听取各民主党派、工商联、无党派人士和人民团体的意见，积极接受新闻舆论和社会公众监督。坚持科学民主依法决策，健全政府重大决策事项程序规则，完善公众参与、专家咨询、风险评估、合法性审查和集体讨论决定的决策机制。坚持公开为原则、不公开为例外，积极推行决策公开、审批公开、信息公开、办事公开。健全行政执法程序，提高行政执法队伍素质，开展依法行政评议考核，切实做到规范公正文明执法。加强政府系统内部层级监督，支持监察、审计等部门依法独立履行监督职责。坚持以人为本。始终保持亲民爱民的公仆情怀，头脑中时刻想着群众，感情上紧紧贴近群众，与人民群众同呼吸、共命运、心连心。始终坚持问政于民、问需于民、问计于民，切实做到重大决策顺民而定，工作措施应民而生，发展绩效为民而求，凡是违背民意的决策坚决不作、损害群众利益的事情坚决不干、劳民伤财的形象工程坚决不上。始终做到勤政为民、务实干事，深入群众、深入基层、深入一线，扎扎实实为民办实事、做好事、解难事。坚持真抓实干。加快永州发展，关键要做实功、干实事、求实效。坚持从永州发展大局出发，紧紧围绕把事干成干好，想办法、出主意、作决策。勇于担责，敢于碰硬，只要是看准了的事，只要是有利于永州发展的事，就要雷厉风行，盯死看牢，一抓到底。深入推进＂四深入两结对一测评＂活动，扎实推进＂一线工作法＂。推行政府绩效考核，加强机关效能建设，优化经济发展环境，严格行政问责制度，大力整治行政慢作为、不作为和乱作为行为。坚持清正廉洁。严格执行党风廉政建设责任制的各项规定，扎实推进政府系统反腐倡廉工作，做到有腐必反，有案必查。加强对政府性资金、重大项目和民生工程的财政、审计和社会监督，强化土地使用、矿山开发、政府采购、工程建设等领域专项治理，完善各项管理制度，切实减少制度漏洞。严格控制＂三公消费＂，切实降低行政成本，树立风正气顺、清正廉洁的良好形象。

各位代表，征程万里风正劲，重任千钧须奋蹄。让我们在省委、省政府和市委的正确领导下，在市人大、市政协的监督支持下，同心同德，奋发图强，为实现科学赶超、开放崛起、富民强市宏伟目标而努力奋斗！
